# Development of Type 1 Diabetes in Wild Bank Voles Associated With Islet Autoantibodies and the Novel Ljungan Virus

**DOI:** 10.1080/15438600303733

**Published:** 2003

**Authors:** Bo Niklasson, Knud E. Heller, Bryan Schønecker, Mogens Bildsøe, Terri Daniels, Christiane S. Hampe, Per Widlund, William T. Simonson, Jonathan B. Schaefer, Terri Daniels, Elizabeth Rutledge, Lynn Bekris, A. Michael Lindberg, Susanne Johansson, Eva Örtqvist, Bengt Persson, Åke Lernmark

**Affiliations:** 1 Apodemus AB Stockholm Sweden; 2 Zoological Institute University of Copenhagen Copenhagen Denmark; 3 Department of Medicine University of Washington R. H. Williams Laboratory 1959 N. E. Pacific Street, Box 357710 Seattle Washington 98195 USA; 4 Department of Chemistry and Biomedical Sciences University of Kalmar Kalmar Sweden; 5 Astrid Lindgrens Children's Hospital Karolinska Institutet Stockholm Sweden

## Abstract

Wild bank voles (*Clethrionomys glareolus*) may develop diabetes in laboratory captivity. The aim of this study was to test whether bank voles develop type 1 diabetes in association with Ljungan virus. Two groups of bank voles were analyzed for diabetes, pancreas histology, autoantibodies to glutamic acid decarboxylase (GAD65), IA-2, and insulin by standardized radioligand-binding assays as well as antibodies to in vitro transcribed and translated Ljungan virus antigens. Group A represented 101 trapped bank voles, which were screened for diabetes when euthanized within 24 hours of capture. Group B represented 67 bank voles, which were trapped and kept in the laboratory for 1 month before being euthanized. Group A bank voles did not have diabetes. Bank voles in group B (22/67; 33%) developed diabetes due to specific lysis of pancreatic islet beta cells. Compared to nondiabetic group B bank voles, diabetic animals had increased levels of GAD65 (*P* < .0001), IA-2 (*P* < .0001), and insulin (*P* = .03) autoantibodies. Affected islets stained positive for Ljungan virus, a novel picorna virus isolated from bank voles. Ljungan virus inoculation of nondiabetic wild bank voles induced beta-cell lysis. Compared to group A bank voles, Ljungan virus antibodies were increased in both nondiabetic (*P* < .0001) and diabetic (*P* = .0015) group B bank voles. Levels of Ljungan virus antibodies were also increased in young age at onset of newly diagnosed type 1 diabetes in children (*P* < .01). These findings support the hypothesis that the development of type 1 diabetes in captured wild bank voles is associated with Ljungan virus. It is speculated that bank voles may have a possible zoonotic role as a reservoir and vector for virus that may contribute to the incidence of type 1 diabetes in humans.


*Experimental Diab. Res.*, 4:35–44, 2003
Copyright © 2003 Taylor & Francis
1543-8600/03 $12.00 + .00
DOI: 10.1080/15438600390214617

Taylor & Francis healthsciences

### Development of Type 1 Diabetes in Wild Bank Voles Associated With Islet Autoantibodies and the Novel Ljungan Virus

**Bo Niklasson,[1] Knud E. Heller,[2] Bryan Schønecker,[2] Mogens Bildsøe,[2] Terri Daniels,[3] Christiane S. Hampe,[3] Per Widlund,[3] William T. Simonson,[3] Jonathan B. Schaefer,[3] Elizabeth Rutledge,[3] Lynn Bekris,[3] A. Michael Lindberg,[4] Susanne Johansson,[4] Eva Örtqvist,[5] Bengt Persson,[5] and Åke Lernmark[3]**

[1]*Apodemus AB, Stockholm, Sweden*
[2]*Zoological Institute, University of Copenhagen, Copenhagen, Denmark*
[3]*Department of Medicine, University of Washington, R. H. Williams Laboratory, Seattle, Washington, USA*
[4]*Department of Chemistry and Biomedical Sciences, University of Kalmar, Kalmar, Sweden*
[5]*Astrid Lindgrens Children's Hospital, Karolinska Institutet, Stockholm, Sweden*

**Wild bank voles (*Clethrionomys glareolus*) may develop diabetes in laboratory captivity. The aim of this study was to test whether bank voles develop type 1 diabetes in association with Ljungan virus. Two groups of bank voles were analyzed for diabetes, pancreas histology, autoantibodies to glutamic acid decarboxylase (GAD65), IA-2, and insulin by standardized radioligand-binding assays as well as antibodies to in vitro transcribed and translated Ljungan virus antigens. Group A represented 101 trapped bank voles, which were screened for diabetes when euthanized within 24 hours of capture. Group B represented 67 bank voles, which were trapped and kept in the laboratory for 1 month before being euthanized. Group A bank voles did not have diabetes. Bank voles in group B (22/67; 33%) developed diabetes due to specific lysis of pancreatic islet beta cells. Compared to nondiabetic group B bank voles, diabetic animals had increased levels of GAD65 ($P < .0001$), IA-2 ($P < .0001$), and insulin ($P = .03$) autoantibodies. Affected islets stained positive for Ljungan virus, a novel picorna virus isolated from bank voles. Ljungan virus inoculation of nondiabetic wild bank voles induced beta-cell lysis. Compared to group A bank voles, Ljungan virus antibodies were increased in both nondiabetic ($P < .0001$) and diabetic ($P = .0015$) group B bank voles. Levels of Ljungan virus antibodies were also increased in young age at onset of newly diagnosed type 1 diabetes in children ($P < .01$). These findings support the hypothesis that the development of type 1 diabetes in captured wild bank voles is associated with Ljungan virus. It is speculated that bank voles may have a possible zoonotic role as a reservoir and vector for virus that may contribute to the incidence of type 1 diabetes in humans.**

Received 18 December 2002; accepted 9 Jaunary 2003.

The authors thank Sue Blaylock and Lisa Hammerle for excellent assistance and A. Notkins, J.-W. Yoon, and H. Foy for helpful comments. This study was supported in part by grants from the Danish Natural Science Research Council, the National Institutes of Health (grant DK26190), the Foundation of Queen Silvia's Jubilee Fund for Research on Children and Handicaps, Förenade Liv Mutual Group Life Insurance Company of Health, and the Swedish Freemason's Foundation. A.M.L. and S.J. were supported from the University of Kalmar and C.S.H. by a fellowship from the Juvenile Diabetes Research Foundation.

Address correspondence to Åke Lernmark, Department of Medicine, University of Washington, R. H. Williams Laboratory, 1959 N. E. Pacific Street, Box 357710, Seattle, WA 98195, USA. E-mail: ake@u.washington.edu

**Keywords** Clethrionomys Glareolus; Glutamic Acid Decarboxylase Autoantibodies; IA-2 Autoantibodies; Insulin Autoantibodies; Insulin-Dependent Diabetes Mellitus; Ljungan Virus; Parechovirus; Picorna Virus

Type 1 (insulin-dependent) diabetes appears after specific destruction of the pancreatic islet beta cells. The degree of beta-cell loss varies with age and is more severe in young children than adults. The disease is strongly associated with beta-cell autoimmunity (for recent reviews see [1, 2]). Autoantibodies against glutamic acid decarboxylase (GAD65), tyrosine phosphatase–like protein IA-2, or insulin, detected in standardized antibody tests [3], predict type 1 diabetes [4]. Monozygotic twins show only 20% to 30% concordance of type 1 diabetes, indicating a significant contribution of environmental factors [5]. This is further supported by the fact that more than 85% of new-onset patients do not have a first-degree relative with the disease [6]. Seasonal variation in incidence rate, together with serological studies, have suggested viral infections as a major environmental risk factor for type 1 diabetes [7]. Congenital rubella [8], as well as infections by enterovirus, in particular during pregnancy [9, 10], are also implicated in type 1 diabetes [11]. Rota virus seroconversion was reported to be associated with increases in autoantibodies to GAD65, IA-2, and insulin, suggesting that infection with this virus may trigger or exacerbate islet autoimmunity in genetically susceptible subjects [12]. However, it is still highly controversial how virus may induce beta-cell destruction. Different hypotheses include direct cytolytic infections resulting in an autoimmune response to released antigen, bystander activation of self-reactive T cells, alterations in target-cell defenses, or molecular mimicry [11, 13, 14]. In the latter hypothesis, the host is infected by a virus that has antigens, which are immunologically similar to the host but different enough to induce a T-cell response.

The lack of animals developing diabetes following lytic infections of beta cells have made it difficult to study these phenomena of potential importance to the etiology of human type 1 diabetes. Type 1 diabetes is known to occur in animals outside research laboratories, but to our knowledge a zoonotic relationship has not been reported. There are numerous zoonoses causing human illness, and bank voles all over the world are well-known reservoirs and vectors for Puumala, leptospirosis, cow pox, and *Borrelia burgdorferi*, which all infect humans [15]. Puumala virus causes nephropathia epidemica [16, 17] and the incidence rate of this disease correlates with the bank vole population density during the previous year [18]. Similar statistical evidence suggests that type 1 diabetes in humans also tracks the 3- to 4-year population density cycles of the bank vole [19]. More recently, wild bank voles taken to the laboratory for studies of "stereotypic" behavior [20] were found to develop polydipsia and glucosuria [21]. In the present study, we provide evidence that bank voles develop type 1 diabetes associated with lytic destruction of their beta cells and increased levels of GAD65, IA-2, and insulin autoantibodies. We also tested the hypothesis that the bank vole diabetes may be induced by Ljungan virus, a newly identified picornavirus [22]. Ljungan virus was isolated from bank voles and shares molecular features with human parechovirus, a known human pathogen [23]. Molecular analyses of several isolates of Ljungan virus suggests that the strains constitute a monophyletic group clearly related to parechovirus of *Picornaviridae* [23, 24].

### MATERIALS AND METHODS

### Bank Voles

Wild bank voles were trapped from May to November in a forest habitat on the island of Zealand, Denmark. Group A bank voles represents 101 animals from a single trapping session. These bank voles were tested within 24 hours after trapping for glucosuria and then euthanized. Heart-blood samples for blood glucose, ketosis, lipids, and antibody analyses were taken immediately after the voles were euthanized. Blood samples were either immediately analyzed for blood glucose and ketones or centrifuged for 25 minutes at $1000 \times g$ and plasma stored at $-30^{\circ}$C.

In the same trapping session, 67 voles were caught and kept in the laboratory as previously described [20, 21]. Briefly, the animals were housed individually in standard laboratory mouse cages under a minimum of extraneous disturbance and with a 12-hour light regime (8.00 to 20.00). The cages were supplied with a woodcutting bed, and food (standard rodent chow) and water was available ad libitum. Diabetes development was determined after a month by measurements of glucosuria, and blood glucose and ketonemia.

### Pancreas Histology and Immunocytochemistry

Standard hematoxylin and eosin staining was carried out on pancreas fixed in 4% paraformaldehyde and embedded in paraffin. Immunocytochemistry was performed as described previously with minor modifications [25]. Briefly, the binding of guinea pig insulin antibodies (diluted 1:100), rabbit glucagon antibodies (Zymed, San Francisco, CA; diluted 1:100), or mouse Ljungan virus antisera (diluted 1:500 or more) to bank vole pancreas tissue was revealed by successive incubations with biotinylated anti–immunoglobulin G (IgG), either alkaline phosphatase streptavidin ABC reagent (Vector Laboratories, Burlingame, CA), or Vectastain peroxidase streptavidin ABC reagent (Vector Laboratories), and either

Vector NBT/BCIP substrate (insulin), Vector DAB substrate (glucagon), or Vector Red substrate (Ljungan virus). High-titer mouse antisera [22] against 2 distinct Ljungan virus isolates, 87-012 and 145SL [23], were used. As control sera we used similarly produced antisera against members of other virus families, such as Bunyavirus (Rift Valley fever), Togavirus (Ockelbo/Sindbis), and Flavivirus (Langat), as well as normal mouse serum. Finally, slides were counter stained with methyl green, dehydrated, and mounted. All slides were coded and scored independently by two readers.

### Islet Cell Autoantibodies

Standardized [3, 26] radioligand-binding assays for autoantibodies against GAD65 [27, 28], IA-2 [29], and insulin [30] autoantibodies were used to analyze serum samples from both bank voles and humans. The World Health Organization (WHO) standard 97/550 was used to express levels of GAD65 and IA-2 autoantibodies in common units [3].

### Ljungan Virus Antibodies

Sera from children with type 1 diabetes and controls were tested for presence of antibodies to Ljungan virus using an indirect immunofluorescence test using spot slides prepared by incubating virus in Green monkey kidney cells for 8 to 10 days. At signs of discrete cytopathic effects, cells were applied onto microscope slides, air dried, fixed in cold (4°C) acetone and stored at −70°C until used. The titer was determined after incubating the serum at several dilutions on the slides and bound antibodies detected with fluorescein isothiocyanate (FITC)-conjugated goat anti-human IgG (Sigma, St Louis, MO). Patient and control sera was first tested at a 1:8 dilution using 3 Ljungan virus isolates (87-012, 145SL, 174F). Any sera scoring positive for any of the 3 isolates were titrated again using all 3 isolates separately. Patient and control sera were scored either as negative (neg) or positive provided that one or several isolates at a titer of 32 (+) or higher (++) were considered positive.

A radioligand-binding assay similar to our GAD65 and IA-2 autoantibody assays [27] was developed with $^{35}$S-labeled virus antigens generated by coupled in vitro transcription and translation of Ljungan virus cDNA [21].

### Type 1 Diabetes Patients and Controls

Two groups of new onset diabetic children were studied. The first group represented a total of 53 children with a median age of 10.1 years (range 2.3 to 16.4 years of age) and were diagnosed with type 1 diabetes at the St Göran Hospital and Astid Lindgren's Children's Hospital, Stockholm, Sweden, between 1992 and 1995. Within 2 days of diagnosis, blood samples were drawn for antibody analyses. Healthy children (7 boys, median age 12.6 [7.8 to 16.8 years] and 10 girls, median age 13.5 [6.7 to 16.6 years]) were recruited from school classes in central Stockholm. All children were previously healthy and without present medication. The second group of children represented 289 children with newly diagnosed type 1 diabetes in 1995 to 2000. A separate group of 37 young adult healthy controls were also examined together with this large group of new onset diabetic children.

The Ethics Committee at the Karolinska Institute, Stockholm, Sweden, approved the study.

### Statistical Analysis

Mann-Whitney test was used for differences in levels. Spearman rank correlation was used to test relationships between antibody levels.

### RESULTS

### Bank Vole Diabetes

The 2 groups of bank voles were analyzed for diabetes, pancreas histology, and autoantibodies against GAD65, IA-2, and insulin as well as antibodies against Ljungan virus. Group A (n = 101) bank voles were euthanized within 24 hours after capture. The average blood glucose in the wild-caught GROUP A bank voles was 101±28 mg/dL (mean ± SD). Four female animals had high blood glucose values of 215, 302, 313, and 343 mg/dL, respectively. These high values remain unexplained because the pancreas histology was normal and there was no glucosuria (data not shown). The results suggest that overt diabetes may not be common or easily detected in the wild.

Group B (n = 67) bank voles were taken to the laboratory after capture and kept in mouse cages for 1 month. We observed that 22/67 (33%) of the group B bank voles had blood glucose above 200 mg/dL, range 211 to 540 mg/dL (Table 1). As many as 18/22 (82%) had ketones, polydipsia, or both. Type 1 diabetes was therefore a likely classification because the bank voles were not only hyperglycemic but also positive for glucosuria, ketonuria, ketonemia, and hyperlipidemia (data not shown). The pancreas histology was examined in all the bank voles to test if a type 1 diabetes classification would be supported by a loss of beta cells and perhaps by insulitis.

### Pancreatic Islet Histology and Immunocytochemistry in Diabetic Bank Voles

The pancreas of the 101 group A bank voles showed normal islets, as did those of nondiabetic group B bank voles (Figure 1*a*, *b*). Immunostaining with insulin and glucagon

**TABLE 1**
The frequency of diabetes in wild-caught and in captive bank voles

| | Group of bank voles | |
|---|---|---|
| | A. Analyzed at trapping | B. Trapped and captive |
| n | 101 | 67 |
| M/F ratio | 42/59 | 29/38 |
| Body weight (g) | 19 ± 5 | 24 ± 4 |
| Blood glucose (mg/dL) | | |
| Nondiabetic | 101 ± 28 | 86 ± 24 |
| Diabetic | None | 346 ± 88 |
| Diabetes, n (%) | 0/101 (0%) | 22 (33%) |
| M/F ratio | N/A | 14/8 |

*Note.* Mean values ± SD are shown. N/A, not applicable.

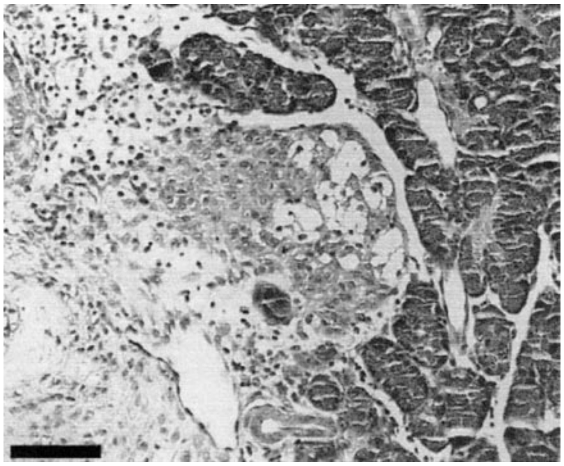

**FIGURE 2**
Histology of the pancreas of a diabetic bank vole demonstrating both lysis of islet cells and some infiltration of mononuclear cells. Hematoxylin and eosin staining. The scale bar is 100 $\mu$m.

antibodies showed a normal islet cell distribution, with beta cells located in the center surrounded by a mantel of glucagon immunoreactive cells (Figure 1*e*, *f*). In dramatic contrast, all 22 group B bank voles with diabetes had a major loss of insulin-positive cells, which were replaced by prominent vacuolization (Figure 1*g*, *h*). Few bank voles had islets with infiltrating mononuclear cells (Figure 2). Mononuclear cell infiltration was not observed in any of the 101 group A or the 45 group B normoglycemic bank voles. Because beta-cell destruction was observed in all diabetic but not in normoglycemic bank voles, we conclude that the bank voles diabetes was consistent with type 1 diabetes.

In order to evaluate whether Ljungan virus was associated with the islet beta-cell lesion, we next immunostained

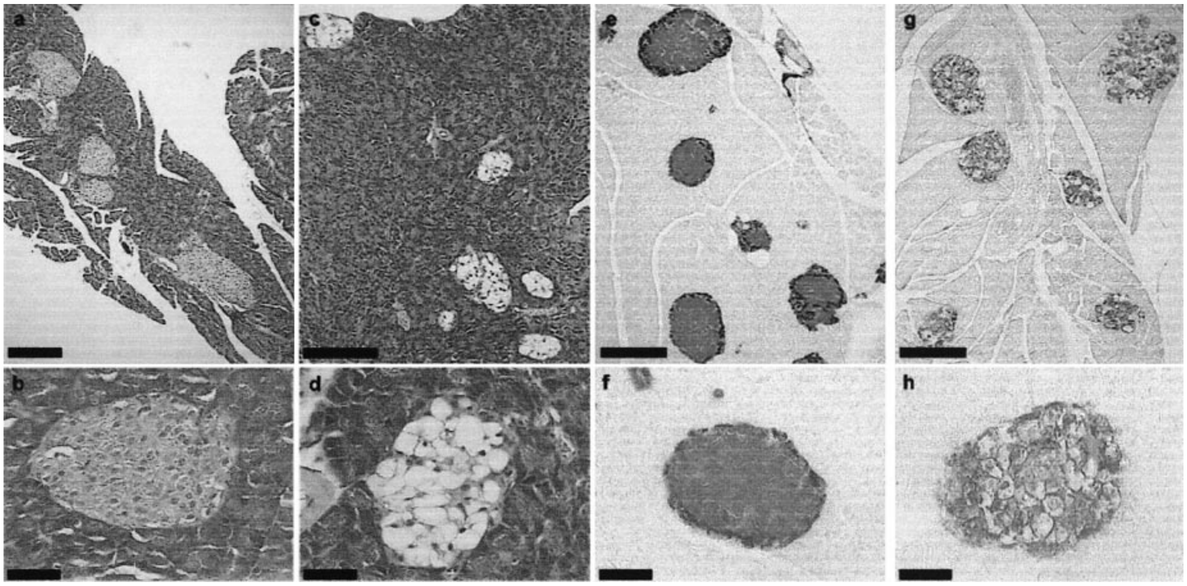

**FIGURE 1**
Histology of the pancreas in bank voles without diabetes (*a*, *b*, *e*, *f*) and with diabetes (*c*, *d*, *g*, *h*). Hematoxylin and eosin staining are shown in *a–d*, while immunostaining for insulin (*blue*) and glucagon (*brown*) are shown in *e–h*. The pancreatic islets in the diabetic bank vole show complete lysis without insulitis. Glucagon cells are found in the periphery of the islets but tend to be redistributed in the pancreatic islets, which show vacuolization after the loss of beta cells. Scale bars: *a*, *c*, *e*, and *g*, 200 $\mu$m; *b*, *d*, *f*, and *h*, 50 $\mu$m.

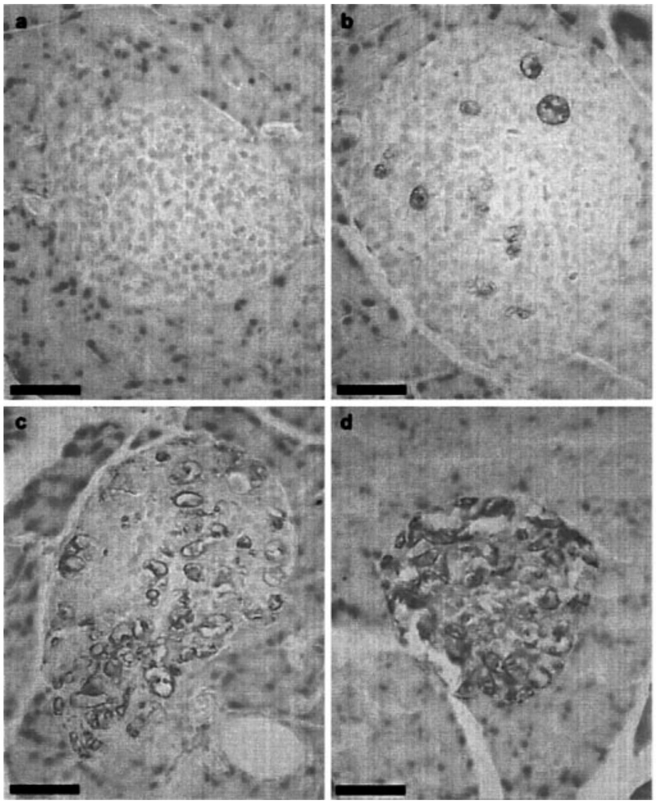

**FIGURE 3**
Histology of the pancreas in a nondiabetic wild bank vole (*a*), nondiabetic bank voles injected with 1000 TCID50 Ljungan virus of the 145SL isolate and then euthanized for histology after 6 weeks (*b*, *c*), and from a diabetic bank vole (*d*). The sections were immunostained with the mouse 145SL antiserum against Ljungan virus. Although the nondiabetic bank vole is negative for Ljungan virus antibody staining, both the virus-inoculated nondiabetic (*b*, *c*) and the diabetic (*d*) bank voles show positive staining at the site of islet lesions. Note the difference in the extent of lysis in the virus inoculated nondiabetic (*b*, *c*) compared to the diabetic bank vole (*d*). The binding of the mouse Ljungan virus antiserum was revealed with Vector Red staining. The scale bar is 100 $\mu$m.

the pancreas sections with high titer mouse antisera against the Ljungan virus isolates, 87-012 and 145SL (Figure 3). The control sera were all negative. Both the 87-012 and the 145SL Ljungan virus antisera at dilutions of 1:4000 or higher immunostained islets in diabetic (Figure 3*d*) but not in nondiabetic bank voles (Figure 3*a*), suggesting that Ljungan virus antigen may be present in affected islets. None of the control sera against Bunyavirus (Rift Valley fever), Togavirus (Ockelbo/Sindbis), and Flavivirus (Langat) in addition to normal mouse serum showed immunostaining at dilutions of 1:500 or higher. We therefore next tested whether the 145SL Ljungan virus would induce beta-cell lesions when injected into wild bank voles. As virus-free, laboratory bank voles were not available, this experiment had to be carried out in group B bank voles kept in the laboratory without developing diabetes.

### Ljungan Virus Inoculation in Nondiabetic Bank Voles

A total of 15 nondiabetic wild bank voles, which failed to developed diabetes in Copenhagen, were transferred to Stockholm and inoculated with saline (n = 5) or the 145SL isolate of the Ljungan virus (n = 10). All animals were normoglycemic when they were euthanized after 6 weeks. The histology of the pancreas was normal in the saline-injected bank voles but there were different degrees of lytic lesions in the islets of the virus-inoculated bank voles (Figure 3*b*, *c*) compared to the pancreas of a diabetic bank vole (Figure 3*d*). The staining pattern with the Ljungan virus mouse antiserum suggests that the beta cell–specific destruction was associated with virus antigen staining. The inoculation with the 145SL Ljungan virus was therefore associated with lytic effects on beta cells. The absence of diabetes may be explained by several factors, such as a short time of follow-up, immunoprotection by neutralizing virus antibodies due to prior Ljungan virus exposure in the wild, or poor virulence of the 145SL isolate. Clearly these experiments are suggestive and will need to be repeated once a virus-free bank vole colony has been established.

### GAD65, IA-2, and Insulin Autoantibodies in Diabetic Bank Voles

The beta-cell destruction was of lytic character (Figures 1 and 3) and surprisingly free from mononuclear cell infiltration (Figure 2). The absence of prominent insulitis supports the view that Ljungan virus is inducing beta-cell lysis that may cause release and presentation of sequestered antigen most likely in lymph nodes draining the pancreas. We therefore examined the possibility that the development of bank vole diabetes was associated with GAD65, IA-2, or insulin autoantibodies.

Group A and group B bank voles were examined for all 3 islet cell autoantibodies (Figure 4). Levels of autoantibodies were examined because an upper level of normal or cut-off could not be established in the absence of virus free wild bank voles. We observed that diabetic group B bank voles had higher GAD65 ($P < .0001$) (Figure 4*a*), IA-2 ($P < .0001$) (Figure 4*b*), and insulin ($P < .03$) (Figure 4*c*) autoantibody levels than the nondiabetic group B bank voles. Nondiabetic group B bank voles also had increased GAD65 ($P < .0001$) (Figure 4*a*) and IA-2 ($P < .0001$) autoantibody

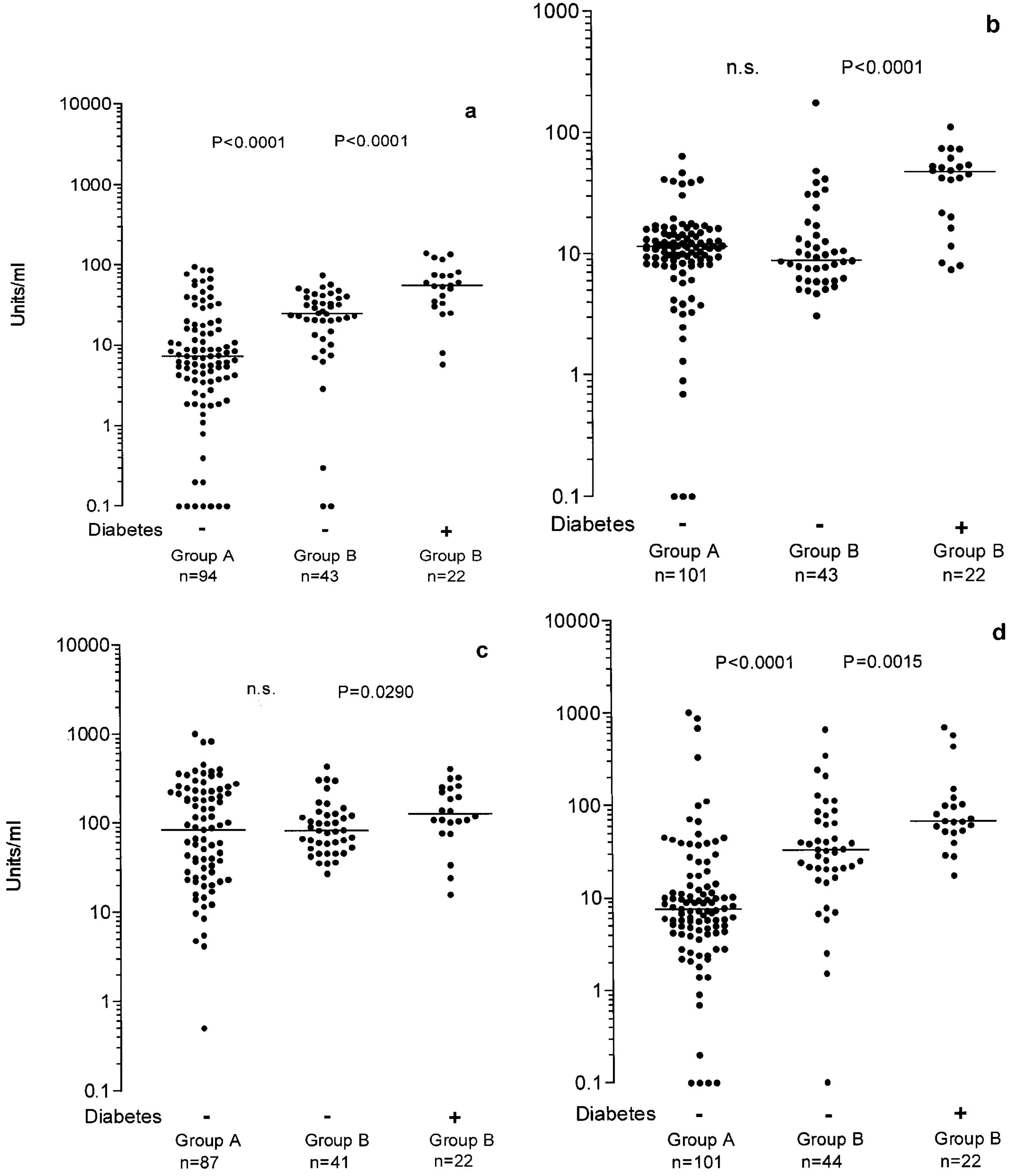

**FIGURE 4**

Bank voles have autoantibodies against islet cell autoantigens and against Ljungan virus in vitro translated antigens. It is indicated whether the bank voles had diabetes (+) or not (−). Autoantibodies to GAD65 (*a*), IA-2 (*b*), insulin (*c*), as well as Ljungan virus in vitro translated antigens (*d*) are shown as in-house relative units on a log scale. Data for individual bank voles are shown along with the median value and *P* values between groups.

levels compared to the group A bank voles, suggesting that bank voles may have been exposed to Ljungan virus without progressing to diabetes, similar to the inoculation experiment described above. The increased levels of GAD65, IA-2, and insulin autoantibodies support the conclusion that diabetes in the group B bank voles should be classified as type 1 diabetes.

### Ljungan Virus Antibodies in Diabetic Bank Voles

Because Ljungan virus antigen was demonstrated in the islets of diabetic bank voles (Figure 3*d*), we next analyzed whether antibodies to Ljungan virus antigens were associated with bank vole diabetes. First, the group A bank vole sera showed a wide range of antibody levels against Ljungan virus antigen (Figure 4*d*), suggesting that some animals had been exposed to the virus in the wild. Note that the *y*-axis of Ljungan virus antigen antibodies shows log values. Second, in the group B bank voles, the levels of Ljungan virus antibodies were higher in diabetic than nondiabetic animals ($P = .0015$).

Because the diabetic group B bank voles also showed increased levels of GAD65, IA-2, and insulin autoantibodies, we next tested if any of the 3 autoantibodies were related to Ljungan virus antibody levels. First, in the diabetic group B voles, Ljungan virus antibodies correlated to GAD65 autoantibodies ($r_s = .4726$, $P < .03$; n = 22), whereas the correlation to IA-2 autoantibodies was suggestive ($P = .052$). Second, in the nondiabetic group B bank voles, Ljungan virus antibodies correlated to autoantibodies against GAD65 ($r_s = .3566$, $P = .02$; n = 43), IA-2 ($r_s = .4171$, $P = .005$; n = 43), as well as to insulin ($r_s = .4389$, $P = .004$; n = 42). In bank voles, it is therefore possible that a Ljungan virus attack on the beta cells is related to an immune response to GAD65, IA-2, or insulin whether or not the bank vole has overt diabetes.

### Ljungan Virus Antibodies in Human Type 1 Diabetes

Similar to other viruses carried by bank voles [15, 16, 31], the Ljungan virus might also infect humans and we therefore tested whether type 1 diabetics and their controls may have Ljungan virus antibodies as detected by indirect immunofluorescence or in a radiobinding assay.

The indirect immunofluorescence virus antibody test was compared to the radioligand-binding assay for Ljungan virus antigen antibodies (Figure 5). There was a significant correlation (Spearman rank sum correlation) between the 2 assays ($P < .0001$, n = 70). Compared to the 17 healthy control children, the 53 children with new onset type 1 diabetes had

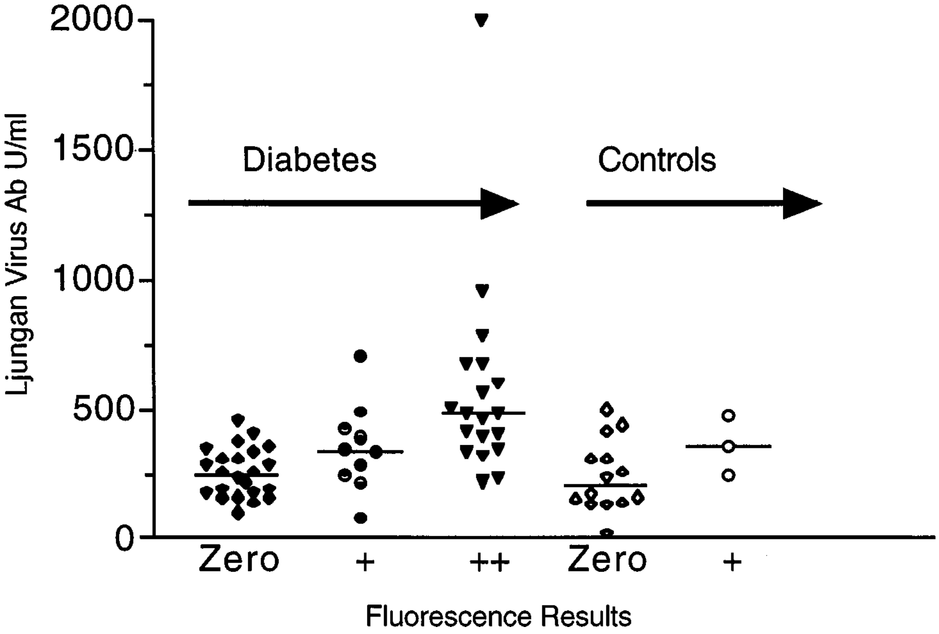

**FIGURE 5**

Children with new onset type 1 diabetes have Ljungan virus antibodies. A total of 53 type 1 diabetes children (•) and 17 controls (○) were tested. Ljungan virus antibodies were determined by either indirect immunofluoresence of cells used to propagate the virus or by the radioligand binding assay with Ljungan virus in vitro translated antigens. Sera from children with type 1 diabetes and controls were tested for presence of antibodies to Ljungan virus using an indirect immunofluorescence test using spot slides prepared by incubating virus in Green monkey kidney cells.

an increased frequency of Ljungan virus antibodies scored in the immunofluorescence assay ($P < .009$) (Figure 5).

A subsequent analysis of 289 newly diagnosed type 1 diabetes children and 37 healthy controls showed that that the levels of Ljungan virus antibodies increased with decreasing age at diagnosis Spearman ($r = -.151$, $P < .01$). These data suggest that children with newly diagnosed type 1 diabetes may have been exposed to Ljungan virus.

### DISCUSSION

Diabetes in bank voles was first indicated during a study of stereotypic behavior in bank voles [20, 21]. In the present study, we found that one third of the wild bank voles trapped alive and brought to the laboratory developed clinical diabetes with hyperglycemia, ketonemia, ketonuria, hyperlipidemia, and weight loss, all criteria that are consistent with a clinical classification of type 1 diabetes [32]. The classification was supported by the observation that the diabetic voles had increased levels of GAD65, IA-2, and insulin autoantibodies detected with the same internationally standardized methods that are used to predict type 1 diabetes in humans [1, 4]. Furthermore, the bank vole diabetes was linked with specific beta-cell destruction and presence of Ljungan virus antigen in affected islets. In addition, the diabetic bank voles had increased levels of antibodies to Ljungan virus antigens compared to the nondiabetic bank voles. We conclude therefore that bank voles caught in the wild and kept in standard mouse cages with food for laboratory mice may spontaneously develop diabetes in association with Ljungan virus infection, which fulfills current criteria for autoimmune type 1 diabetes. The Ljungan virus, a newly identified picorna-like virus [22] may therefore be the etiological factor causing type 1 diabetes in the bank vole. This is a novel finding that may be of considerable significance because it is the first time a wild rodent is shown to develop type 1 diabetes in association with beta-cell lysis without insulitis, but with all 3 autoantibodies associated with human type 1 diabetes.

In the present study, we showed that infecting nondiabetic wild bank voles could induce beta-cell lesions. As pointed out, we cannot exclude that the inoculation of the 145SL Ljungan virus isolate accelerated beta-cell destruction by a virus already present in the wild bank vole. Attempts are in progress to breed and generate specific pathogen–free bank voles to expose them to currently available Ljungan virus isolates [23]. Inoculation of various strains of mice is also of interest but may prove futile because our experience so far failed to induce diabetes when raising mouse antibodies against the current Ljungan virus isolates (Niklasson, unpublished observations).

The histology of the islets in the diabetic voles suggests that the beta cells were destroyed and replaced by vacuoles or possibly fatty infiltration. The less common occurrence of mononuclear cell infiltrated islets differs from what is reported in, e.g., Coxsackie virus–infected mice [33, 34]. The lack of widespread insulitis, however, did not preclude the animals from developing autoantibodies against GAD65, IA-2, and insulin, which suggests that an autoimmune response to these autoantigens is not dependent on insulitis. The location of antigen presentation is much debated, but data in the BDC2.5 T-cell receptor transgenic nonobese diabetic (NOD) mouse suggest that beta-cell antigens are specifically transported to pancreatic lymph nodes, where they trigger reactive T cells that invade the islets [35]. This suggestion was supported by studies of Coxsackie B4 virus–induced diabetes in laboratory mice, which demonstrated that the primary role of virus is to damage the beta cells to cause release and presentation of sequestered islet antigen [14]. In humans, it is not surprising that the initial lesion is difficult to study because most of the beta-cell destruction has already taken place once type 1 diabetes is diagnosed. The long prodrome is reflected by the fact that islet cell autoantibodies may be detected years before diagnosis [1, 4]. Nevertheless, several reports of diabetes occurring close to Coxsackie [36, 37] or echo 9 [38] virus infections have been reported, which suggest that in some patients infection may still lead to a beta-cell destruction and diabetes. Although further studies will be required, our data would support the hypothesis that beta-cell destruction causes release and presentation of sequestered beta-cell antigens [14]. Furthermore, a complete depletion of antigen may preclude a subsequent infiltration of antigen-presenting cells and T lymphocytes of the type that is seen in inbred, spontaneously diabetic NOD mice (reviewed by [2]). These and other questions will be possible to answer once a virus-free colony of bank voles has been established. Such animals will also permit an analysis of cut-off levels of autoantibodies, which could not be accomplished in the present study of wild bank voles.

Our result that the Ljungan virus antibodies in the diabetic bank voles correlated with GAD65 antibody was not surprising because high-titer animal antisera against coxsackie virus also react with GAD65 [39]. It is important to point out that the virus antigen prepared by in vitro transcription translation fulfill all the criteria to detect conformation-dependent autoantibodies [4], but may not detect linear epitopes of virus-specific antigens. Additional tests may be necessary to establish the diagnostic sensitivity and specificity for Ljungan virus immunity. In addition, it cannot be excluded that antibody-binding sites that are common to Ljungan and other Parechoviruses are detected in the present system. Our radioligand-binding antibody assay may therefore be able to discriminate immune from nonimmune sera on a population basis, but perhaps not on an individual basis. The antibody results in the bank voles are, however, consistent with the hypothesis that the Ljungan virus is the etiologic agent responsible for beta cell destruction in the bank voles. The human serological data are also very intriguing, but additional studies using a variety of serological tests as well as polymerase chain reaction (PCR) detection of virus will be needed to establish a relationship between Ljungan virus infection and human type 1 diabetes. Although the mechanism of such a process remains unclear, our initial studies of diabetic bank voles suggest that stress might be involved in diabetes development [20, 21]. Stress has also been implicated in human type 1 diabetes because negative life events increase the risk for childhood type 1 diabetes [40–42]. Similar relationships could be relevant to our bank voles and therefore aid in understanding of zoonotic infections from bank voles.

Taken together, we have demonstrated the following. First, bank voles develop diabetes that fulfills the criteria for type 1 diabetes: diabetic animals showed persistent hyperglycemia associated with weight loss, ketosis, and hyperlipidemia and specific beta-cell destruction. Second, diabetic voles had increased levels of autoantibodies to GAD65, IA-2, and insulin, and these autoantibodies correlated with Ljungan virus antigen antibodies. Third, the association between Ljungan virus and bank vole diabetes was supported by the presence of Ljungan virus antigen detected by immunocytochemistry in the islets of diabetic bank voles. Finally, increased levels of Ljungan virus antibodies in newly diagnosed type 1 diabetes children indicate a possible zoonotic relationship between Ljungan virus infection and human type 1 diabetes.

### REFERENCES

[1] Atkinson, M. A., and Eisenbarth, G. S. (2001) Type 1 diabetes: New perspectives on disease pathogenesis and treatment. *Lancet,* **358,** 221–229.

[2] Mathis, D., Vence, L., and Benoist, C. (2001) Beta-cell death during progression to diabetes. *Nature,* **414,** 792–798.

[3] Mire-Sluis, A. R., Das, R. G., and Lernmark, Å. (2000) The World Health Organization International Collaborative Study for Islet Cell Antibodies. *Diabetologia,* **43,** 1282–1292.

[4] Notkins, A. L., and Lernmark, A. (2001) Autoimmune type 1 diabetes: Resolved and unresolved issues. *J. Clin. Invest.,* **108,** 1247–1252.

[5] Kyvik, K. O., Green, A., and Beck, N. H. (1995) Concordance rates of insulin dependent diabetes mellitus: A population based study of young Danish twins [see comments]. *BMJ,* **311,** 913–917.

[6] Dahlquist, G., Blom, L., Tuvemo, T., Nyström, L., Sandström, A., and Wall, S. (1989) The Swedish Childhood Diabetes Study—results from a nine year case register and one year case-referent study indicating that type 1 (insulin-dependent) diabetes mellitus is associated with both type 2 (non-insulin-dependent) diabetes mellitus and autoimmune disorders. *Diabetologia,* **32,** 2–6.

[7] Dahlquist, G. G. (1997) Viruses and other perinatal exposures as initiating events for beta-cell destruction. *Ann. Med.,* **29,** 413–417.

[8] Menser, M. A., Forrest, J. M., and Bransby, R. D. (1978) Rubella infection and diabetes mellitus. *Lancet,* **i,** 57–60.

[9] Dahlquist, G., Ivarsson, S., Lindberg, B., and Forsgren, M. (1995) Maternal enteroviral infection during pregnancy as a risk factor for childhood IDDM. *Diabetes,* **44,** 408–413.

[10] Hyöty, H., Hiltunen, M., Knip, M., Laakkonen, M., Vahasalo, P., Karjalainen, J., Koskela, P., Roivainen, M., Leinikki, P., and Hovi, T. (1995) A prospective study of the role of coxsackie B and other enterovirus infections in the pathogenesis of IDDM. Childhood Diabetes in Finland (DiMe) Study. *Diabetes,* **44,** 652–657.

[11] Knip, M., and Akerblom, H. K. (1999) Environmental factors in the pathogenesis of type 1 diabetes mellitus. *Exp. Clin. Endocrinol. Diabetes,* **107,** S93–S100.

[12] Honeyman, M. C., Coulson, B. S., Stone, N. L., BGellert, S. A., Goldwater, P. N., Steele, C. E., Couper, J. J., Tait, B. D., Colman, P. G., and Harrison, L. C. (2000) Association between rotavirus infection and pancreatic islet autoimmunity in children at risk of developing type 1 diabetes. *Diabetes,* **49,** 1319–1324.

[13] Flodstrom, M., Maday, A., Balakrishna, D., Cleary, M. M., Yoshimura, A., and Sarvetnick, N. (2002) Target cell defense prevents the development of diabetes after viral infection. *Nat. Immunol.,* **3,** 373–382.

[14] Horwitz, M. S., Ilic, A., Fine, C., Rodriguez, E., and Sarvetnick, N. (2002) Presented antigen from damaged pancreatic b cells activates autoreactive T cells in virus-mediated autoimmune diabetes. *J. Clin. Invest.,* **109,** 79–87.

[15] Hazel, S. M., Bennett, M., Chantrey, J., Bown, K., Cavanagh, R., Jones, T. R., Baxby, D., and Begon, M. (2000) A longitudinal study of an endemic disease in its wildlife reservoir: Cowpox and wild rodents. *Epidemiol. Infect.,* **124,** 551–562.

[16] Myhrman, G. (1934) En njursjukdom med egenartad symptombild. *Nordisk Medicinsk Tidskrift,* **7,** 739–794.

[17] Niklasson, B., and Le Duc, J. (1984) Isolation of the nephropathia epidemica agent in Sweden. *Lancet,* **1,** 1012–1013.

[18] Niklasson, B., Hornfeldt, B., Lundkvist, A., Bjorsten, S., and Leduc, J. (1995) Temporal dynamics of Puumala virus antibody prevalence in voles and of nephropathia epidemica incidence in humans. *Am. J. Trop. Med. Hyg.,* **53,** 134–140.

[19] Niklasson, B., Hornfeldt, B., and Lundman, B. (1998) Could myocarditis, insulin-dependent diabetes mellitus, and Guillain-Barre syndrome be caused by one or more infectious agents carried by rodents? *Emerg. Infect. Dis.,* **4,** 187–193.

[20] Schoenecker, B., and Heller, K. E. (2000) Indication of a genetic basis of stereotypies in laboratory-bred bank voles (*Clethrionomys glareolus*). *Appl. Anim. Behav. Sci.,* **68,** 339–347.

[21] Johansson, S., Niklasson, B., Maizel, J., Gorbalenya, A. E., and Lindberg, A. M. (2002) Molecular analysis of three Ljungan virus isolates reveals a new, close-to-root lineage of the Picornaviridae with a cluster of two unrelated 2A proteins. *J. Virol.,* **76,** 8920–8930.

[22] Lindberg, A. M., and Johansson, S. (2002) Phylogenetic analysis of Ljungan virus and A-2 plaque virus, new members of the Picornaviridae. *Virus Res.,* **85,** 61–70.

[23] Schoenecker, B., Heller, K. E., and Freimanis, T. (2000) Development of sterotypies and polydipsia in wild caught bank voles (*Clethrionomys glareolus*) and their laboratory-bred offspring. Is polydipsia a symptom of diabetes mellitus? *Appl. Anim. Behav. Sci.,* **68,** 349–357.

[24] Lee, E. S., Jiang, J., Sund, G. C., Simonson, W. T., Graham, J., Dietsch, G., Schimpf, B., Bieg, S., Peterman, G., and Lernmark, A. (1999) Recombinant human platelet-activating factor acetylhydrolase reduces the frequency of diabetes in the diabetes-prone BB rat. *Diabetes,* **48,** 43–49.

[25] Niklasson, B., Kinnunen, L., Hornfeldt, B., Horling, J., Benemar, C., Hedlund, K. O., Matskova, L., Hyypia, T., and Winberg, G. (1999) A new picornavirus isolated from bank voles (*Clethrionomys glareolus*). *Virology,* **255,** 86–93.

[26] Verge, C. F., Stenger, D., Bonifacio, E., Colman, P. G., Pilcher, C., Bingley, P. J., and Eisenbarth, G. S. (1998) Combined use of autoantibodies (IA-2 autoantibody, GAD autoantibody, insulin autoantibody, cytoplasmic islet cell antibodies) in type 1 diabetes: Combinatorial Islet Autoantibody Workshop. *Diabetes,* **47,** 1857–1866.

[27] Grubin, C. E., Daniels, T., Toivola, B., Landin-Olsson, M., Hagopian, W. A., Li, L., Karlsen, A. E., Boel, E., Michelsen, B., and Lernmark, Å. (1994) A novel radioligand binding assay to determine diagnostic accuracy of isoform-specific glutamic acid decarboxylase antibodies in childhood IDDM. *Diabetologia,* **37,** 344–350.

[28] Hampe, C. S., Hammerle, L. P., Bekris, L., Ortqvist, E., Kockum, I., Rolandsson, O., Landin-Olsson, M., Torn, C., Persson, B., and Lernmark, A. (2000) Recognition of glutamic acid decarboxylase (GAD) by autoantibodies from different GAD antibody-positive phenotypes. *J. Clin. Endocrinol. Metab.,* **85,** 4671–4679.

[29] Kawasaki, E., Yu, L., Gianani, R., Verge, C. F., Babu, S., Bonifacio, E., and Eisenbarth, G. S. (1997) Evaluation of islet cell antigen (ICA) 512/IA-2 autoantibody radioassays using overlapping ICA512/IA-2 constructs. *J. Clin. Endocrinol. Metab.,* **82,** 375–380.

[30] Williams, A. J. K., Bingley, P. J., Bonifacio, E., Palmer, J. P., and Gale, E. A. M. (1997) A novel micro-assay for insulin autoantibodies. *J. Autoimmun.,* **10,** 473–478.

[31] Niklasson, B., and LeDuc, J. W. (1987) Epidemiology of nephropathia epidemica in Sweden. *J. Infect. Dis.,* **155,** 269–276.

[32] Committee, A. E. (1997) Report of the Expert Committee on the Diagnosis and Classification of Diabetes Mellitus. *Diabetes Care,* **20,** 1183–1197.

[33] Rayfield, E. J., and Ishimura, K. (1987) Environmental factors and insulin-dependent diabetes mellitus. *Diabetes Metab. Rev.,* **3,** 925–957.

[34] Yoon, J. W. (1995) A new look at viruses in type 1 diabetes. *Diabetes Metab. Rev.,* **11,** 83–107.

[35] Höglund, P., Mintern, J., Waltzinger, C., Heath, W., Benoist, C., and Mathis, D. (1999) Initiation of autoimmune diabetes by developmentally regulated presentation of islet cell antigens in the pancreatic lymph nodes. *J. Exp. Med.,* **189,** 331–339.

[36] Gladisch, R., Hofmann, W., and Waldherr, R. (1976) Myokarditis und Insulitis nach Coxsackie-Virus-Infekt. *Kardiologie,* **65,** 835–849.

[37] Yoon, J.-W., Austin, M., Onodera, T., and Notkins, A. L. (1975) Isolation of a virus from the pancreas of a child with diabetic ketoacidosis. *N. Engl. J. Med.,* **300,** 1174–1179.

[38] Vreugdenhil, G. R., Schloot, N. C., Hoorens, A., Rongen, C., Pipeleers, D. G., Melchers, W. J., Roep, B. O., and Galama, J. M. (2000) Acute onset of type I diabetes mellitus after severe echovirus 9 infection: Putative pathogenic pathways. *Clin. Infect. Dis.,* **31,** 1025–1031.

[39] Hou, J., Said, C., Franchi, D., Dockstader, P., and Chatterjee, N. K. (1994) Antibodies to glutamic acid decarboxylase and P2-C peptides in sera from Coxsackie virus B4-infected mice and IDDM patients. *Diabetes,* **43,** 1260–1266.

[40] Dahlquist, G., Blom, L., and Lönnberg, G. (1991) The Swedish Childhood Diabetes Study—a multivariate analysis of risk determinants for diabetes in different age groups. *Diabetologia,* **34,** 757–762.

[41] Hägglöf, B., Blom, L., Dahlquist, G., Lonnberg, G., and Sahlin, B. (1991) The Swedish childhood diabetes study: Indications of severe psychological stress as a risk factor for type 1 (insulin-dependent) diabetes mellitus in childhood. *Diabetologia,* **34,** 579–583.

[42] Thernlund, G. M., Dahlquist, G., Hansson, K., Ivarsson, S. A., Ludvigsson, J., Sjoblad, S., and Hagglof, B. (1995) Psychological stress and the onset of IDDM in children. *Diabetes Care,* **18,** 323–329.

